# Exploring turnover among first-line managers in healthcare: a cohort study of span of control, management performance and stress indicators

**DOI:** 10.1108/LHS-02-2025-0031

**Published:** 2025-06-23

**Authors:** Jonas Svanström, Maria Lindberg, Bernice Skytt, Magnus Lindberg

**Affiliations:** Department of Caring Sciences, Faculty of Health and Occupational Studies, University of Gävle, Gavle, Sweden; Department of Caring Science, Faculty of Health and Occupational Studies, University of Gävle, Gavle, Sweden, and Department of Public Health and Caring Sciences, Faculty of Medicine, Uppsala University, Uppsala, Sweden; Department of Caring Sciences, Faculty of Health and Occupational Studies, University of Gävle, Gavle, Sweden

**Keywords:** Control, First-line managers, Healthcare, Managerial complexity, Stress, Turnover

## Abstract

**Purpose:**

The purpose of this study is to examine if and how an expanded span of control, management performance and work-related stress indicators (control, support and relationships) influence the time until first-line managers leave their position.

**Design/methodology/approach:**

A prospective longitudinal cohort design involving 87 first-line managers in a Swedish health-care region. Cox proportional hazards regression was used to analyse expanded span of control, work-related stress indicators and management performance as predictors of turnover.

**Findings:**

The findings indicate that first-line managers who were categorized as having moderate concern and a need for improvement in the work-related stress indicators control and manager support had a significantly higher risk of turnover. In contrast, expanded span of control and the number of employees managed per first-line manager did not significantly influence turnover risk. In addition, the work-related stress indicator relationships was not significantly associated with risk of turnover.

**Originality/value:**

This study expands the understanding of actual turnover among first-line managers in health care by exploring how organizational factors influence the decision to leave. Unlike previous research, which primarily examines turnover intentions, this study takes a longitudinal perspective, capturing real turnover events over time. The findings contribute to future research on organizational conditions, providing a basis for developing strategies to improve retention and working conditions for first-line managers in health care.

## Introduction

Turnover among first-line managers in health care is a significant challenge, driven by a combination of organizational, professional and personal factors (Labrague, 2020). These challenges often stem from the complexities inherent in their role, such as balancing personnel management with financial oversight and fostering a positive work environment while contending with resource constraints, administrative burdens and ethical dilemmas that can contribute to moral distress (Edwin *et al.*, 2023). Moreover, in their role, first-line managers might need to disrupt established routines and act swiftly and collaboratively to respond to sudden unknown and changing contexts such as the COVID-19 pandemic (Hartney *et al.*, 2021). A systematic review by Penconek *et al.* (2024) highlights that first-line managers frequently have moderate levels of autonomy and get limited support from their managers, which leaves them feeling only partially empowered and often overwhelmed by their responsibilities. A study by Niinihuhta *et al.* (2022) indicated that first-line managers generally report high work-related well-being. However, nearly half describe their work as often or always challenging, with high job demands acting as significant stressors. In addition, Svanström *et al.* (2025) found moderate to strong correlations indicating that greater control over work and managerial support were associated with higher job satisfaction, whereas high demands and strained relationships were associated with lower job satisfaction. These results underscore the critical need for supportive work environments to reduce stress and improve retention among first-line managers. The impact of stress is particularly pronounced in high-pressure environments like teaching hospitals and acute care facilities, where insufficient resources and unmanageable workloads exacerbate stress and emotional exhaustion (Hewko *et al.*, 2015; Wong *et al.*, 2015). It is common that first-line managers report moderate to high levels of workplace stress (Jäppinen *et al.*, 2022). Without sufficient support, such stress can escalate, negatively affecting managers’ health (Udod *et al.*, 2017) and job satisfaction, and increasing their desire to leave their role (Cregård and Corin, 2019).

The prevalence of turnover intentions among first-line managers is alarming. Research shows that nearly half of these managers plan to leave their role within three to five years, with many citing burnout, insufficient respect and inadequate support as the primary reasons (Warden *et al.*, 2021; Warshawsky *et al.*, 2016). Burnout alone is identified as a significant driver, reflecting the intense demands placed on this role. However, first-line managers who experience fair workloads, constructive relationships with managers and supportive workplace cultures report higher job satisfaction and lower turnover intentions (Warshawsky *et al.*, 2016). This highlights the importance of fostering positive work environments, as job satisfaction can serve as a buffer against the aforementioned challenges, reducing the likelihood of turnover among first-line managers (Labrague, 2020). Having support from managers and team members has been shown to enhance job satisfaction and reduce turnover intention among first-line managers (Adriaenssens *et al.*, 2017).

Most studies are on turnover intentions, but Skagert *et al.* (2012) provided valuable insights into actual turnover rates, revealing that 40% of Swedish health-care managers with staff and budget responsibilities left their position over a four-year period. The study highlighted job control and overall health as critical factors for retention. Managers who experienced sufficient autonomy in their role and maintained personal well-being were more likely to remain in their position. These findings underscore the necessity of fostering a supportive work environment that promotes autonomy and well-being among health-care managers, as this can significantly increase both retention and overall job satisfaction.

High turnover among first-line managers in health care impacts not only the managers themselves but also the teams they manage. As shown in the study by Akgerman and Sönmez (2020), first-line managers are important to fostering organizational commitment and stability within teams. The findings indicate that nurses’ trust in first-line managers is closely linked to their own commitment to the organization, particularly in terms of affective commitment, which reflects an emotional attachment to their role. First-line managers who establish a supportive, positive work environment and cultivate trust within their teams contribute to increased commitment and reduced turnover intentions among nursing staff. In addition, a high turnover rate among first-line managers is costly for the organization due to the expenses associated with new recruitment (Phillips *et al.*, 2018).

Despite extensive research on factors influencing first-line managers’ intentions to leave a role in health care, there is limited research examining their departure. The limited existing research on actual turnover has primarily focused on multilevel managerial populations, which may obscure the specific challenges and stressors affecting first-line managers. This gap highlights the need for an exploration of how organizational factors and work-related stress indicators might contribute to actual turnover among first-line managers. The aim of this study was to decrease this gap by examining if and how an expanded span of control, management performance and work-related stress indicators (control, manager support and relationships) influence the time until first-line managers leave their position.

## Methods

### Study design

This study used a prospective longitudinal cohort design to examine how an expanded span of control, work-related stress indicators and management performance influence the time until first-line managers leave their position. The STROBE checklist (Elm *et al.*, 2007) was used to guide the reporting of this study.

### Study sample

The study was conducted in a Swedish region’s health-care sector, encompassing primary care, pre-hospital services and inpatient care. Invitations to participate were sent via email to all first-line managers in the region, along with a link to the online survey. To encourage participation, two reminder emails were sent to those who had not responded. The cohort consisted of 87 first-line managers who completed a baseline survey at the start of the study in September 2020. These participants were prospectively monitored in regard to turnover over a period of 40 months. As this study is part of a doctoral education, the follow-up period was determined by the timeframe available within the education. The demographic and employment characteristics of the first-line managers included in the cohort are summarized in Table 1.

**Table 1.** Characteristics of the first-line managers and their status after 40 months

Characteristics	Total in cohort (*n* = 87)	Remained as first-line manager (*n* = 59)	Left role as first-line manager (*n* = 28)
Age Mean (95% CI^b^) and SD^c^	50.1 (48.0–52.2) 9.8	50.8 (48.3–53.1) 9.6	48.6 (44.7–52.5) 10.1
Total years as manager Mean (95% CI^b^) and SD^c^	7.9 (6.5–9.4) 6.8	7.8 (6.2–9.5) 6.3	8.2 (5.1–11.3) 7.9
Number of employees Mean (95% CI) and SD	32.0 (30.1–33.9) 9.0	31.5 (29.2–33.7) 8.7	32.2 (29.5–36.9) 9.6
Months to leave^a^ Mean (95% CI) and SD	–	–	21.9 (18.5–25.2) 8.6

**Note(s):**

^a^ = Months to leave is measured from the start of the study until the participant left the role as a first-line manager;

^b^ = confidence interval;

^c^ = standard deviation

**Source(s):** Authors’ own work

### Measures

The main outcome variable was turnover, defined as the event of a first-line manager leaving their position during the study period. These data were provided from the region’s human resources department. The variables included in this study were selected to provide an organizational perspective on turnover among first-line managers, focusing on expanded span of control, management performance and work-related stress indicators.

Expanded span of control was measured using The Ottawa Hospital Model of Nursing Clinical Practice and Clinical Management Span of Control Decision-Making Indicators (TOH-SOC) (Morash *et al.*, 2005). This is a validated tool (Morash *et al.*, 2005) that evaluates managerial complexity across three key domains: unit-focused, staff-focused and programme-focused. The unit-focused domain includes factors such as operational hours, patient turnover and risk management, as well as time required for equipment maintenance and vendor interactions. The staff-focused domain assesses the number of direct reports, staff skill levels, turnover rates, absenteeism and the balance between novice and experienced staff, reflecting the level of managerial support needed. The programme-focused domain examines broader responsibilities, including budgetary oversight, service diversity and management of multiple units. Together, these domains provide a comprehensive framework for assessing the complexities of first-line managerial responsibilities. A score between 61 and 90 represents an appropriate span of control, whereas a score between 91 and 130 indicates an excessive span of control, suggesting that additional support may be required.

Management performance was assessed using the Leadership and Management Inventory (LaMI-II), a validated instrument (Skytt *et al.*, 2023). The full inventory consists of 25 items divided into two subscales, leadership and management performance. Only the management performance subscale was used in this study. This subscale evaluates skills in planning, organizing and ensuring regulatory compliance. Each item is rated on a five-point Likert scale, ranging from “not at all” to to“a very large extent”. Scores for management performance range from 20 to 100, with reliability metrics indicating high internal consistency, as shown in Table 2.

**Table 2.** Model 1 predictors of turnover among first-line managers

Variable	Df^a^	Exp(B)	95% CI^b^ (lower–upper)	*p*-value	Cronbach’s alpha
TOH-SOC^c^					
Expanded span of control	1	0.986	0.941–1.033	0.543	
LaMI-II^d^					
Management	1	1.042	0.990–1.096	0.119	0.86
HSE^e^					
Control					0.79
Green^f^ (≥80th percentile)	3			0.078	
Aqua^g^ (50th–80th percentile)	1	0.771	0.265–2.249	0.634	
Yellow^h^ (20th–50th percentile)	1	6.576	1.274–33.953	0.025^*^	
Red^i^ (<20th percentile)	1	1.375	0.525–3.604	0.517	
Manager support					0.87
Green^f^ (≥80th percentile)	3			0.148	
Aqua^g^ (50th–80th percentile)	1	0.000	0.000–0.000	0.974	
Yellow^h^ (20th–50th percentile)	1	3.876	1.122–13.395	0.032^*^	
Red^i^ (<20th percentile)	1	0.831	0.147–1.657	0.716	
Relationships	1	1.375	0.525–3.604	0.517	0.72

**Note(s):**

* = significant at 0.05.

^a^ = degrees of freedom.

^b^ = confidence interval.

^c^ = The Ottawa Hospital Model of Nursing Clinical Practice and Clinical Management Span of Control Decision-Making Indicators.

^d^ = Leadership and Management Inventory.

^e^ = Health and Safety Executive Management Standards Indicator Tool.

^f^ = high performance with no immediate action needed.

^g^ = good performance with room for improvement.

^h^ = moderate concern and a need for improvement.

^i^ = low performance, requiring urgent action

**Source(s):** Authors’ own work

Work-related stress was measured using selected indicators of the Health and Safety Executive Management Standards Indicator Tool (HSE) (Cousins *et al.*, 2004). The HSE questionnaire is a validated instrument (Edwards *et al.*, 2008). We used three subscales in the questionnaire: control, manager support and relationships. The control indicator evaluates employees’ autonomy in their work, including their ability to decide when to take breaks, set their work pace and influence how tasks are completed. The manager support indicator assesses the feedback, assistance and emotional support that managers provide. The relationships indicator examines workplace interactions, including exposure to personal harassment, bullying, friction and strained relationships between colleagues. Ratings of control and manager support were categorized in accordance with the tool guidelines (Cousins *et al.*, 2004): green (≥80th percentile) indicates high performance with no immediate action needed, aqua/blue (50th–80th percentile) reflects good performance with room for improvement, yellow (20th–50th percentile) signifies moderate concern and a need for improvement and red (<20th percentile) indicates low performance, requiring urgent action (Table 3). This was not possible for the indicator relationships, which could not be grouped as 84 out of 87 participants rated themselves in the same category. Therefore, it was retained as a continuous variable.

**Table 3.** Colour coding cut-offs for HSE indicators

Indicators	Red	Yellow	Aqua	Green
	< 20th percentile	>= 20th and < 50th percentile	>= 50th and < 80th percentile	>= 80th percentile
Control	< 3.2240	>= 3.2240 and < 3.4741	>= 3.4741 and < 3.7208	>= 3.7208
Manager support	< 3.2720	>= 3.2720 and < 3.4603	>= 3.4603 and < 3.6500	>= 3.6500

**Source(s):** Authors’ own work

### Statistical analysis

Descriptive statistics were used to present turnover rates among first-line managers over time, expressed as proportions (%) and numbers (*n*). The age distribution of the participants, including mean, confidence interval (CI) and standard deviation (SD), is presented in Table 1. The relationship between turnover and the independent variables was analysed using Cox proportional hazards regression. To mitigate multicollinearity, we ensured that the correlation did not exceed 0.3 between any of the variables included in the two models. The results were expressed as Exp(B), representing the exponentiated coefficient and indicating the relative risk for turnover associated with each predictor variable. Statistical significance was set at *p* < 0.05. All analyses were conducted using IBM SPSS Statistics for Windows, Version 27.0 (IBM Corp, Armonk, New York).

Multiple imputations were applied to handle missing values in this data set. This method involves creating multiple estimates for missing values to reduce bias and enhance the accuracy of analyses (Norman and Streiner, 2014). Multiple imputation is a commonly used technique in statistical analysis, as it allows the retention of the full data set’s integrity by filling in plausible values based on observed data patterns. Missing data for the total score were observed in 3.4% (*n* = 3) of TOH-SOC assessments and 1.1% (*n* = 1) of LaMI-II management assessments. The remaining variables had no missing values. Before performing the imputations, we conducted Little’s missing completely at random (MCAR) test to determine whether the missing data were random or systematic. Little’s MCAR test returned a chi-square statistic of 35.891 with 26 degrees of freedom (DF) and a significance level (*p*-value) of 0.094. As the *p*-value was above the conventional threshold of 0.05, it suggested that the missing data were not significantly different from a random distribution. This indicated that the missing values were likely missing at random, supporting the use of multiple imputations as a suitable method to handle the missing data without introducing bias. With this confirmation, we proceeded with the multiple imputations, filling in plausible values based on observed data patterns to maintain the integrity and completeness of the data set.

In Model 1, we included expanded span of control (TOH-SOC), management performance (LaMI-II) and the work-related stress factor (HSE) indicators control, manager support and relationships. In Model 2, we examined whether the traditional way of measuring span of control differs from the expanded approach. Thus, the variable number of employees per first-line manager replaced the expanded span of control in the equation.

## Ethical considerations

This study was conducted in accordance with the Declaration of Helsinki and received ethical approval from the Swedish Ethical Review Authority, registered under number 2019-04583. At the start of the study, participants were informed that their participation was voluntary and that they could withdraw from the study at any time without explanation.

## Results

During the study period of 40 months, 32.2% (*n* = 28) of the first-line managers in the cohort left their position. The analysis in Model 1 (see Table 2) identified both significant predictors of turnover and variables without significant associations. Expanded span of control was not a significant predictor of turnover among first-line managers (Exp(B) = 0.986). This suggests that the breadth of managerial responsibilities, such as overseeing multiple units or managing diverse staff compositions, does not substantially influence the likelihood of turnover. Similarly, management performance (LaMI-II) did not emerge as a significant predictor of turnover, with an Exp(B) of 1.042.

In contrast, work-related stress indicators revealed significant findings. The control indicator ratings, which measure managers’ autonomy over decision-making and work pace, indicated moderate concern and a need for improvement. This categorized rating was associated with a significantly higher likelihood of turnover (Exp(B) = 6.576). Similarly, the manager support indicator, which evaluates the feedback, assistance and emotional support provided by managers, showed that first-line managers who fell into the category moderate concern and a need for improvement faced a significantly higher risk of turnover (Exp(B) = 3.876). Finally, the relationships indicator, which evaluates workplace interactions and interpersonal tensions, did not significantly predict turnover, with an Exp(B) of 1.375.

In Model 2 (Table 4), the number of employees managed by a first-line manager was not a significant predictor of turnover, with an Exp(B) of 0.999. This suggests that the size of the team does not substantially influence the likelihood of turnover within 40 months. Consistent with the findings in Model 1, the indicators control and manager support from the HSE were significant predictors of turnover. The first-line managers who were categorized as having moderate concern and a need for improvement of the indicator control, such as limited ability to influence how tasks are completed or set their own work pace, faced a higher risk of turnover (Exp(B) = 6.592). Similarly, the manager support indicator indicated moderate concern and a need for improvement. The first-line managers who were categorized in this category had significantly higher likelihood of turnover (Exp(B) = 3.422). The relationships indicator, which evaluates workplace interactions and interpersonal tensions, remained non-significant in Model 2, with an Exp(B) of 1.316.

**Table 4.** Model 2 predictors of turnover among first-line managers

Variable	df^a^	Exp(B)	95% CI^b^ (lower–upper)	*p*-value	Cronbach’s alpha
Number of employees	1	0.999	0.955–1.045		
LaMI-II^c^					
Management	1	1.038	0.988–1.091	0.119	0.86
HSE^d^					
Control					0.79
Green^e^ (≥80th percentile)	3			0.082	
Aqua^f^ (50th–80th percentile)	1	0.781	0.269–2.271	0.650	
Yellow^g^ (20th–50th percentile)	1	6.592	1.262–34.435	0.025*	
Red^h^ (<20th percentile)	1	1.361	0.514–3.603	0.535	
Manager support					0.87
Green^e^ (≥80th percentile)	3			0.169	
Aqua^f^ (50th–80th percentile)	1	0.000	0.000–0.000	0.974	
Yellow^g^ (20th–50th percentile)	1	3.422	1.066–10.991	0.039*	
Red^h^ (<20th percentile)	1	0.822	0.302–2.234	0.700	
Relationships	1	1.316	0.678–2.555	0.417	0.72

**Note(s):**

* = significant at 0.05.

^a^ = degrees of freedom.

^b^ = confidence interval.

^c^ = Leadership and Management Inventory.

^d^ = Health and Safety Executive Management Standards Indicator Tool.

^e^ = high performance with no immediate action needed.

^f^ = good performance with room for improvement.

^g^ = moderate concern and a need for improvement.

^h^ = low performance, requiring urgent action

**Source(s):** Authors’ own work

## Discussion

The aim of this study was to examine if and how an expanded span of control, management performance and work-related stress influence the time until first-line managers leave their position. Our findings indicate that first-line managers’ turnover could be driven by insufficient control over their work and inadequate managerial support.

The first-line managers who were categorized as having moderate concern and a need for improvement in the control indicator were at significantly higher risk of turnover in our study. These findings align with Skagert *et al.* (2012), who identified that moderate to high levels of control were positively associated with remaining in a managerial position over time. Similarly, Labrague (2020) highlighted that insufficient control significantly predicted turnover intention among first-line managers, reinforcing the importance of autonomy in managerial roles. In a qualitative study by (Anonymous *et al.*, Unpublised article), first-line managers described how excessive responsibilities and lack of organizational support create a sense of losing control over workload and work conditions, leading to stress. Findings from a separate qualitative study conducted during the first wave of the pandemic emphasize that first-line managers should prioritize their emotional well-being and foster a supportive work environment to keep the health-care system running (Hartney *et al.*, 2021). Collectively, these findings suggest that control operates along a spectrum, where insufficient control acts as a push factor contributing to turnover. In contrast, adequate levels of control serve as a stabilizing factor that promotes retention. This underscores the importance of providing first-line managers with sufficient autonomy and influence over their work to decrease the turnover risk among first-line managers.

Similarly, first-line managers who were categorized as having moderate concern and a need for improvement in the indicator managerial support had a higher likelihood of turnover in our study. This is in line with (Dolinta and Freysteinson, 2023) integrative review, which emphasized the significance of management support as a predictor of turnover intention. Their findings showed that first-line managers who did not perceive adequate support from their managers had significantly greater intentions to leave their role. This aligns with the findings of Adriaenssens *et al.* (2017) and Keith *et al.* (2021), who emphasized that constructive feedback and emotional support were critical for reducing stress and enhancing job satisfaction among first-line managers. Building on these findings, recent cross-sectional research indicates that job satisfaction increases when first-line managers perceive support from their managers (Svanström *et al.*, 2025). First-line managers value recognition for their leadership efforts and expect support from their managers (Cregård and Corin, 2019; Nagle *et al.*, 2021). However, such support is sometimes perceived as inadequate, whether it is from managers and hospital administration (Nagle *et al.*, 2021). Our findings extend this evidence by demonstrating that managerial support is not just a buffer against stress but also a decisive factor in retention among first-line managers over time. Furthermore, insufficient support leaves many first-line managers feeling overwhelmed, which remains a critical driver of turnover (Penconek *et al.*, 2024). A study by Lundin *et al.* (2024) highlights the importance of managerial support as an important structural condition for effective leadership. Their findings indicate that first-line managers who receive guidance, recognition and advocacy from their managers feel more secure and empowered in their roles. Conversely, a lack of such support increases stress and weakens the ability to lead. Moreover, managerial support plays a crucial role in reinforcing legitimacy and trust within the organization, factors essential for long-term retention. These insights align with our results, highlighting the need for health-care organizations to strengthen managerial support to reduce turnover among first-line managers.

Interestingly, our study found that neither expanded span of control nor the number of employees managed by first-line managers significantly predicted turnover. An integrative review by Boned-Galán *et al.* (2023) highlighted that a wider span of control can increase role overload and weaken leadership effectiveness. However, its impact depends on factors such as leadership experience, institutional support and access to administrative resources. Supporting this perspective, Miller and Hemberg (2023) found that first-line managers responsible for more than 25 employees experienced increased workload demands and a greater need for support. However, they also emphasized that workload is not solely determined by team size, as leadership support and staff characteristics also play a crucial role. This aligns with our findings, where the indicators control and manager support, rather than expanded span of control or number of employees, were significant predictors of turnover. Further emphasizing the complexity of span of control, a study by Hagerman *et al.* (2016) showed that municipal first-line managers with fewer than 30 subordinates experienced greater autonomy in decision-making and perceived themselves as having stronger formal and informal authority than those managing larger teams. These findings suggest that although span of control alone may not predict turnover, it can influence first-line managers’ perceived ability to lead effectively. Having sufficient resources and decision-making authority is essential for maintaining leadership capacity and reducing stress, which in turn may impact retention. This aligns with our findings, where control over work and managerial support, rather than the number of employees managed, were significant predictors of turnover.

First-line managers’ self-rated management performance was not a significant predictor of turnover, suggesting that performance related to planning, organizing and ensuring regulatory compliance alone does not determine whether first-line managers leave their role. These findings indicate that factors beyond individual management performance play a greater role in first-line managers’ decisions to leave. Previous research describes that first-line managers often operate in demanding frontline environments where immediate operational duties take priority over strategic involvement (Gjøsæter and Kyvik, 2021). Despite being expected to engage in strategizing, their ability to do so is frequently hindered by unpredictable day-to-day challenges, administrative burdens and limited support from upper management. Furthermore, financial constraints restrict their autonomy, particularly in public organizations, making it difficult to influence decision-making (Gjøsæter and Kyvik, 2021). It is important that first-line managers receive adequate support when they enter their role. Research highlights that first-line managers who do not receive sufficient guidance and training from the start are more likely to experience stress and consider leaving their position (Loveridge, 2017). Without proper support, the demands of the role might become overwhelming.

Another noteworthy finding is that the relationships indicator, including aspects like interpersonal tensions and interactions, was not a significant predictor of turnover in our study. Previous studies have described that first-line managers who experience poor relationships with employees and superiors, including unsupportive or negative behaviour, cite these challenges as factors contributing to their decision to leave the role (Anonymous *et al.*, Unpublised article 2; Tuna and Kahraman, 2019). Whereas previous research has found correlations between positive relationships and higher job satisfaction (Svanstrom *et al.*, n.d., Warshawsky *et al.*, 2016), our findings suggest that relationships do not play a decisive role in predicting turnover among first-line managers. This indicates that although workplace relationships may shape overall work experiences, other factors, such as autonomy and managerial support, have a more direct impact on turnover decisions.

## Limitations

This study has certain limitations that could influence the generalizability and validity of its findings. The sample size of 87 first-line managers, limited to one region in Sweden, restricts the transferability of results to other regions or health-care systems. The study’s emphasis on organizational factors, such as workload and stress, does not account for personal variables. In addition, the measurement tools used, such as TOH-SOC and LaMI-II, focus on specific aspects of managerial roles and may not fully capture their complexity. Another limitation is the relatively low number of events in relation to predictors. With 32 recorded events across five variables in the models, the events-per-variable ratio is 6.4, which is below the commonly recommended threshold of ≥10 to ensure robust regression estimates (Ogundimu *et al.*, 2016). However, an events-per-variable ratio of ≥5 can still yield acceptable results. Although the low events-per-variable ratio may increase the risk of model instability (Ogundimu *et al.*, 2016), this study still provides valuable insights into turnover among first-line managers.

## Conclusion

This study highlights that insufficient control over work tasks and inadequate managerial support can be drivers of turnover among first-line managers in Swedish health care. The turnover rate was 32.2% over 40 months. To improve retention, it is important that health-care organizations focus on strengthening managerial support and providing first-line managers with greater autonomy in their role. By addressing these factors, organizations can create sustainable working conditions that empower first-line managers and enhance their ability to lead effectively. The findings emphasize the need for targeted interventions to reduce turnover and support sustainable leadership. Future research should explore additional factors contributing to turnover and develop strategies tailored to the unique challenges faced by first-line managers in the health-care sector.
